# Incidental Cavitary Pulmonary Mycobacterium avium Complex Disease With Possible Haemophilus influenzae Coinfection and Pharmacologic Barriers to Therapy: A Case Report

**DOI:** 10.7759/cureus.112143

**Published:** 2026-07-06

**Authors:** Robert Alhaddadin, Amal Qsous, Parth Bhagat, Ekeoma Onuoha, Jocelyn Zusurekuu, Palaniandy Kogulan, Nicholas Haddad

**Affiliations:** 1 Internal Medicine, Covenant HealthCare College of Medicine, Central Michigan University, Saginaw, USA; 2 Internal Medicine, University of Debrecen Medical School, Debrecen, HUN; 3 Infectious Diseases, Covenant HealthCare College of Medicine, Central Michigan University, Saginaw, USA; 4 Infectious Disease, Internal Medicine, Covenant HealthCare College of Medicine, Central Michigan University, Saginaw, USA

**Keywords:** apixaban, case report, cavitary lung disease, drug-drug interaction, haemophilus influenzae, mycobacterium avium complex, nontuberculous mycobacteria, qt prolongation

## Abstract

Pulmonary disease caused by *Mycobacterium avium* complex (MAC) most often occurs in patients with structural lung disease or immunocompromising conditions and typically follows recognized fibrocavitary or nodular-bronchiectatic radiographic patterns. Advanced cavitary disease discovered incidentally in an asymptomatic patient represents an uncommon and diagnostically challenging presentation that may be initially attributed to malignancy, aspiration pneumonia, or endemic fungal infection. We report the case of a 45-year-old man without known immunodeficiency who had alcohol use disorder and active deep vein thrombosis treated with apixaban and presented with acute right groin pain secondary to a reducible inguinal hernia. Abdominal computed tomography performed during hernia evaluation incidentally demonstrated a 6.9 × 5.3 cm thick-walled cavitary opacity in the left lower lobe with an air-fluid level and bilateral reticulonodular opacities. The patient denied cough, dyspnea, hemoptysis, fever, night sweats, and weight loss. Serial sputum acid-fast bacillus smears were positive, and mycobacterial cultures grew MAC. In the setting of compatible cavitary imaging, these findings fulfilled the American Thoracic Society/European Respiratory Society/European Society of Clinical Microbiology and Infectious Diseases/Infectious Diseases Society of America (ATS/ERS/ESCMID/IDSA) diagnostic criteria for pulmonary MAC disease. Tuberculosis and HIV testing were negative. A respiratory molecular panel was also positive for *Haemophilus influenzae*. Because bacterial cultures showed no growth, this finding was interpreted as a possible coinfection rather than a confirmed bacterial infection. Given the leukocytosis and bilateral infiltrates, intravenous ceftriaxone was initiated empirically. However, MAC-directed multidrug therapy was complicated by two pharmacologic barriers: the risk of reduced apixaban exposure with rifabutin-containing therapy and a baseline corrected QT interval of 512 ms, limiting routine macrolide use. The patient was therefore transferred to a tertiary care center for multidisciplinary regimen selection. This case underscores that incidental cavitary pulmonary lesions demand systematic microbiologic evaluation regardless of symptom burden and that early pharmacologic risk assessment is essential when standard MAC therapy is constrained.

## Introduction

*Mycobacterium avium* complex (MAC) is the most frequently isolated nontuberculous mycobacterium (NTM) associated with pulmonary disease in North America and Western Europe [1,2]. Current international guidelines emphasize that the diagnosis of NTM pulmonary disease requires compatible clinical, radiographic, and microbiologic findings; therefore, isolation of MAC from respiratory specimens should not be interpreted as clinically significant without the appropriate clinical context [1,2].

Two predominant clinico-radiographic patterns are recognized. Fibrocavitary disease classically occurs in older men with pre-existing structural lung disease, whereas nodular-bronchiectatic disease is more often described in older, slender women without a smoking history [1-5]. In contrast, an incidentally discovered large cavitary lesion in a patient without respiratory symptoms represents an atypical and underrecognized presentation that may lead to misclassification or delayed diagnosis.

Recognizing this presentation is clinically important because cavitary lesions require timely differentiation from tuberculosis and malignancy, appropriate interpretation of microbiologic findings supports antimicrobial stewardship, and standard MAC therapy may be constrained by clinically significant drug-drug interactions.

Concurrent bacterial detection may further complicate the interpretation of cavitary lung disease. *Haemophilus influenzae* is an established airway pathogen in chronic obstructive pulmonary disease (COPD) and other conditions associated with impaired mucociliary clearance; however, detection by multiplex molecular testing may represent infection, colonization, or residual nucleic acid depending on the clinical context [6,7]. We describe a 45-year-old man without known immunodeficiency who was found to have incidental cavitary MAC pulmonary disease with concomitant molecular detection of *H. influenzae*. His management was complicated by active anticoagulation and baseline QT prolongation. This case was previously presented as a poster at the Michigan Infectious Disease Society (MIDS) Annual Spring Conference on March 21, 2026. The case is reported in accordance with the CARE case report guidelines [8].

## Case presentation

A 45-year-old man with alcohol use disorder and a history of deep vein thrombosis (DVT) on therapeutic apixaban presented to the emergency department with acute right groin pain. Physical examination revealed a reducible right inguinal hernia, and his groin pain resolved after manual reduction. The patient was afebrile and hemodynamically stable, with no hypoxemia or respiratory distress.

**Figure 1:**
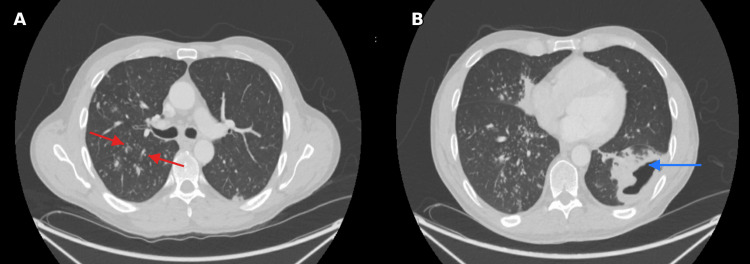
Chest computed tomography findings Axial contrast-enhanced chest computed tomography images. Panel A demonstrates reticulonodular opacities, with red arrows highlighting representative right-sided nodular opacities. Panel B demonstrates a 6.9 × 5.3 cm thick-walled cavitary opacity in the left lower lobe with an internal air-fluid level, highlighted by the blue arrow. No lymphadenopathy or pleural effusion is identified.

Computed tomography (CT) of the abdomen and pelvis with intravenous contrast was obtained as part of the hernia evaluation. Lower thoracic views incidentally revealed a 6.9 × 5.3 cm thick-walled cavitary opacity in the left lower lobe with an air-fluid level, as well as bilateral reticulonodular opacities involving the right upper and lower lobes. A subsequent dedicated chest CT better characterized the cavitary lesion and bilateral reticulonodular opacities (Figure 1).

A focused review of respiratory and constitutional symptoms was negative for cough, sputum production, dyspnea, hemoptysis, pleuritic chest pain, fever, chills, night sweats, and unintentional weight loss at presentation and during the preceding months.

His past medical history was notable for a penetrating left hemithoracic stab wound approximately eight years earlier, complicated by hemopneumothorax requiring tube thoracostomy. Follow-up imaging after that event reportedly demonstrated complete radiographic resolution. The patient had no known history of COPD, bronchiectasis, prior tuberculosis, HIV infection, solid organ or hematopoietic stem cell transplantation, chronic corticosteroid or biologic immunomodulator exposure, or primary immunodeficiency. Family history was negative for chronic lung disease, immunodeficiency, or mycobacterial infection. He reported current tobacco and alcohol use and had no documented illicit substance use.

Given the cavitary pulmonary findings, an infectious disease consultation was obtained. Three sputum specimens collected on separate days showed acid-fast bacilli on direct smear, and subsequent mycobacterial cultures grew MAC. QuantiFERON-TB Gold and HIV-1/2 antigen-antibody testing were negative. In the setting of cavitary imaging with bilateral nodular opacities and repeated positive sputum mycobacterial cultures, these findings fulfilled the American Thoracic Society/European Respiratory Society/European Society of Clinical Microbiology and Infectious Diseases/Infectious Diseases Society of America (ATS/ERS/ESCMID/IDSA) diagnostic criteria for pulmonary MAC disease [1,2].

A respiratory multiplex molecular panel was positive for *H. influenzae*, although sputum bacterial cultures and two sets of blood cultures showed no growth. Laboratory evaluation revealed mild leukocytosis with neutrophilic predominance and an AST-predominant transaminase elevation attributed to alcohol use disorder. Serum albumin was within the reference range. Fungitell testing and QuantiFERON-TB Plus were negative. Additional immunologic testing reviewed by the infectious disease team was documented as unrevealing. Electrocardiography revealed a corrected QT interval (QTc) of 512 ms using the Bazett formula. Selected laboratory and diagnostic test results with corresponding ranges are summarized in Table 1.

**Table 1:** Selected laboratory and diagnostic test results Abbreviations: QTc, corrected QT interval; TB, tuberculosis

Parameter	Patient value	Reference range	Interpretation
White blood cell count	11.41 × 10³/μL	3.40-11.00 × 10³/μL	Mild leukocytosis
Neutrophils	84.00%	44.0-85.0%	Neutrophilic predominance
Absolute neutrophil count	9.58 × 10³/μL	1.50-9.35 × 10³/μL	Mildly elevated
Albumin	3.5 g/dL	3.4-4.8 g/dL	Within reference range
Aspartate aminotransferase	142 U/L	3-48 U/L	Elevated
Alanine aminotransferase	71 U/L	15-71 U/L	Upper limit of reference range
Fungitell quantitative value	<31 pg/mL	<60 pg/mL	Negative
QuantiFERON-TB Plus	Negative	Negative	Negative
Corrected QT interval, Bazett	512 ms	<450 ms in men	Prolonged

Given the leukocytosis and bilateral radiographic infiltrates, intravenous ceftriaxone 2 g every 24 hours was initiated empirically for possible *H. influenzae* coinfection despite the absence of confirmatory culture growth. Initiation of MAC-directed multidrug therapy was deferred because of two clinically significant pharmacologic barriers. First, rifamycin therapy raised concern for reduced apixaban exposure through CYP3A4 and P-glycoprotein induction, with potential loss of therapeutic effect in a patient being treated for DVT [9]. Second, a baseline QTc exceeding 500 ms limited routine macrolide use, particularly azithromycin, given its FDA Drug Safety Communication warning regarding potentially fatal cardiac arrhythmias [10]. Given these competing treatment-limiting factors, the patient was transferred to a tertiary care center for multidisciplinary evaluation by infectious disease, pulmonology, and clinical pharmacology. At the receiving center, after multidisciplinary risk-benefit assessment, he was started on azithromycin, rifabutin, and ethambutol with close monitoring of the QTc interval and anticoagulation status. The sequence of key clinical events is summarized in Table 2.

**Table 2:** Clinical timeline Abbreviations: AFB, acid-fast bacillus; CT, computed tomography; DVT, deep vein thrombosis; IV, intravenous; MAC, *Mycobacterium avium *complex; QTc, corrected QT interval

Time Point	Key Event
Presentation	The patient presented with acute right groin pain due to a reducible right inguinal hernia. His pain resolved after manual reduction. He was afebrile, hemodynamically stable, and had no hypoxemia or respiratory distress.
Imaging	Initial abdominal/pelvic CT: incidentally demonstrated a 6.9 × 5.3 cm thick-walled left lower-lobe cavitary opacity with an air-fluid level and bilateral reticulonodular opacities on lower thoracic cuts. Dedicated chest imaging: subsequent chest CT with IV contrast further characterized the left lower-lobe cavitary lesion and bilateral reticulonodular opacities.
Symptom review	Focused respiratory and constitutional review was negative for cough, sputum production, dyspnea, hemoptysis, pleuritic chest pain, fever, chills, night sweats, and unintentional weight loss at presentation and during the preceding months.
Diagnostic workup	Infectious disease consultation obtained; serial sputum AFB smears and mycobacterial cultures initiated; QuantiFERON-TB Gold and HIV testing were performed.
Microbiology	Three sputum AFB smears were positive on separate days, and mycobacterial cultures grew MAC. QuantiFERON-TB Gold and HIV-1/2 antigen-antibody testing were negative.
Bacterial detection	The respiratory multiplex molecular panel was positive for *H. influenzae*; sputum bacterial cultures and two blood culture sets showed no growth.
Cardiac evaluation	Electrocardiography demonstrated a QTc of 512 ms (Bazett formula).
Initial treatment	Ceftriaxone 2 g IV every 24 hours was initiated empirically for possible *H. influenzae *coinfection, given the leukocytosis and bilateral infiltrates, although bacterial cultures showed no growth. MAC-directed multidrug therapy was deferred pending pharmacologic risk assessment.
Disposition	The patient was transferred to a tertiary care center for multidisciplinary evaluation (infectious disease, pulmonology, clinical pharmacology), given rifabutin-apixaban interaction risk and QTc prolongation limiting macrolide use.

## Discussion

This case illustrates three clinically important issues: the imperative to investigate incidental cavitary lung disease regardless of symptom burden, the interpretive challenge posed by concurrent molecular detection of *H. influenzae*, and the pharmacologic complexity that arises when standard MAC therapy is simultaneously constrained by active anticoagulation and baseline QT prolongation.

Incidental Cavitary MAC Disease Without Respiratory Symptoms

The absence of respiratory symptoms was diagnostically striking given the size and cavitary morphology of the lesion. Importantly, the ATS/ERS/ESCMID/IDSA diagnostic criteria for nontuberculous mycobacterial (NTM) pulmonary disease do not require a specific symptom threshold; rather, they integrate radiographic abnormalities, repeated positive microbiological evidence, and exclusion of alternative diagnoses [1,2]. When a thick-walled cavity with an air-fluid level and bilateral reticulonodular opacities is identified, a broad differential should be considered, including pulmonary tuberculosis; primary or metastatic malignancy; aspiration-related necrotizing pneumonia; endemic fungal infections, such as histoplasmosis, coccidioidomycosis, and blastomycosis; lung abscess; and NTM disease. This case reinforces that incidental cavitary findings should not be dismissed solely because respiratory or constitutional symptoms are absent. In some patients, minimal symptom burden may reflect disease chronicity or physiologic adaptation rather than limited disease severity.

*Host Factors and Phenotypic Considerations* 

The patient did not fit the classic profile for fibrocavitary MAC disease. He was younger than the typical affected population and had no known COPD, bronchiectasis, prior tuberculosis, or immunosuppressive medication exposure. Alcohol use disorder may have contributed to pulmonary susceptibility through aspiration risk, impaired mucociliary clearance, altered innate immune function, and nutritional vulnerability, although it is not a well-established independent risk factor for pulmonary MAC disease [11]. A remote left-sided hemopneumothorax was considered as a possible structural contributor, but reported radiographic resolution after that event made it unlikely to fully account for the current cavitary disease. Immunologic testing was unrevealing. 

Interpretation of Concurrent H. influenzae Molecular Detection

The positive *H. influenzae *molecular result required careful contextual interpretation. Multiplex respiratory molecular panels can detect organisms not recoverable by standard culture, particularly fastidious bacteria susceptible to transport delays or prior antibiotics. However, molecular detection alone does not establish causation; *H. influenzae* can colonize the upper airway, persist as residual nucleic acid, or be detected in chronic airway disease in the absence of active infection [6,7]. In this patient, the combination of leukocytosis with neutrophilia and bilateral infiltrates supported empiric treatment for possible *H. influenzae *coinfection, whereas the repeatedly positive acid-fast bacillus (AFB) smears and MAC culture growth established MAC as the primary diagnosis. Because bacterial cultures showed no growth, the *H. influenzae* finding could not be confirmed as bacterial pneumonia. Interpreting the finding as a possible coinfection more accurately reflects the available data and has direct implications for antimicrobial stewardship and ceftriaxone duration.

*Pharmacologic Barriers to Standard MAC Therapy* 

Standard treatment for cavitary MAC pulmonary disease typically includes a macrolide, ethambutol, and a rifamycin, with parenteral amikacin considered in selected patients with severe or cavitary disease [1]. In this patient, regimen construction was complicated by two major safety concerns. First, rifamycin therapy posed a clinically significant interaction risk with apixaban. Rifampin is a strong inducer of CYP3A4 and P-glycoprotein and can substantially reduce apixaban exposure, with potential loss of anticoagulant effect in a patient being treated for DVT [9]. Although rifabutin has a less potent induction profile than rifampin, the interaction remained clinically relevant because maintaining therapeutic anticoagulation was clinically important. Transition to low-molecular-weight heparin or warfarin with close monitoring was considered a potential strategy, but required individualized assessment. Second, macrolide therapy was limited by a baseline QTc of 512 ms. Azithromycin is often preferred over clarithromycin in MAC regimens because of better tolerability and fewer drug-drug interactions, but it may increase the risk of ventricular arrhythmia in patients with preexisting QT prolongation [10]. Because both rifabutin use and macrolide therapy carry substantial risk, standard MAC therapy could not be initiated safely without specialist input. The patient was therefore transferred for multidisciplinary evaluation and individualized regimen selection. After discussion of the risks and benefits, azithromycin, rifabutin, and ethambutol were initiated with close monitoring of the QTc interval and anticoagulation status. 

Limitations

This report has several limitations. The *H. influenzae* finding was based on molecular testing without confirmatory culture growth; therefore, active coinfection could not be definitively distinguished from colonization or clinically relevant detection. In addition, MAC susceptibility testing, regimen adherence, culture-conversion outcomes, and short- and long-term clinical follow-up after transfer, including treatment tolerance and clinical response, were unavailable at the time of this report. These limitations restrict conclusions regarding the role of *H. influenzae* and the patient's subsequent treatment course.

## Conclusions

Incidental cavitary pulmonary lesions require systematic microbiologic evaluation even when respiratory and constitutional symptoms are absent. In this patient, repeated positive AFB smears and MAC cultures, together with compatible cavitary imaging and negative tuberculosis testing, satisfied established diagnostic criteria for pulmonary MAC disease. Concurrent molecular detection of *H. influenzae *supported empiric treatment for possible coinfection but required cautious interpretation because bacterial cultures were negative. This case also highlights the importance of early pharmacologic review, as rifamycin-apixaban interaction risk and baseline QT prolongation may substantially limit standard MAC therapy and require individualized multidisciplinary management.
